# Telomeric Repeat-Containing RNA (TERRA): A Review of the Literature and First Assessment in Cutaneous T-Cell Lymphomas

**DOI:** 10.3390/genes13030539

**Published:** 2022-03-18

**Authors:** Alain Chebly, Joana Ropio, Lyla Baldasseroni, Martina Prochazkova-Carlotti, Yamina Idrissi, Jacky Ferrer, Chantal Farra, Marie Beylot-Barry, Jean-Philippe Merlio, Edith Chevret

**Affiliations:** 1 BoRdeaux Institute of Oncology (BRIC), UMR1312, INSERM, University of Bordeaux, 146 Rue Leo Saignat, 33076 Bordeaux, France; alain.chebly@usj.edu.lb (A.C.); joana.ropio@gmail.com (J.R.); lyla.baldasseroni@univ-amu.fr (L.B.); martina.carlotti@u-bordeaux.fr (M.P.-C.); yamina.idrissi@u-bordeaux.fr (Y.I.); jacky_ferrer@yahoo.fr (J.F.); marie.beylot-barry@chu-bordeaux.fr (M.B.-B.); jp.merlio@u-bordeaux.fr (J.-P.M.); 2 Medical Genetics Unit, Faculty of Medicine, Saint Joseph University, Beirut 1104 2020, Lebanon; chantal.farra@usj.edu.lb; 3 Institute of Biomedical Sciences of Abel Salazar, Porto University, 4050-313 Porto, Portugal; 4 Faculty of Veterinary Medicine, Lusófona University, 1749-024 Lisbon, Portugal; 5 Department of Genetics, Hotel Dieu de France Medical Center, Beirut 166830, Lebanon; 6 Department of Dermatology, Bordeaux University Hospital, 33075 Bordeaux, France; 7 Tumor Bank and Tumor Biology Laboratory, Bordeaux University Hospital, 33600 Pessac, France

**Keywords:** TERRA, non-coding RNA, telomere, telomerase, cutaneous T-cell lymphomas, sézary syndrome

## Abstract

Telomeric Repeat-containing RNA (TERRA) are long non-coding RNAs transcribed from telomeric DNA sequences from multiple chromosome ends. Major research efforts have been made to understand TERRA roles and functions in several physiological and pathological processes. We summarize herein available data regarding TERRA’s roles in human cells and we report the first investigation in cutaneous T-cells lymphomas (CTCL) using real-time PCR. Among the TERRA analysed, our data suggest a particular role for TERRA 16p downregulation and TERRA 11q upregulation in CTCL lymphomagenesis.

## 1. Introduction

**Figure 1:**
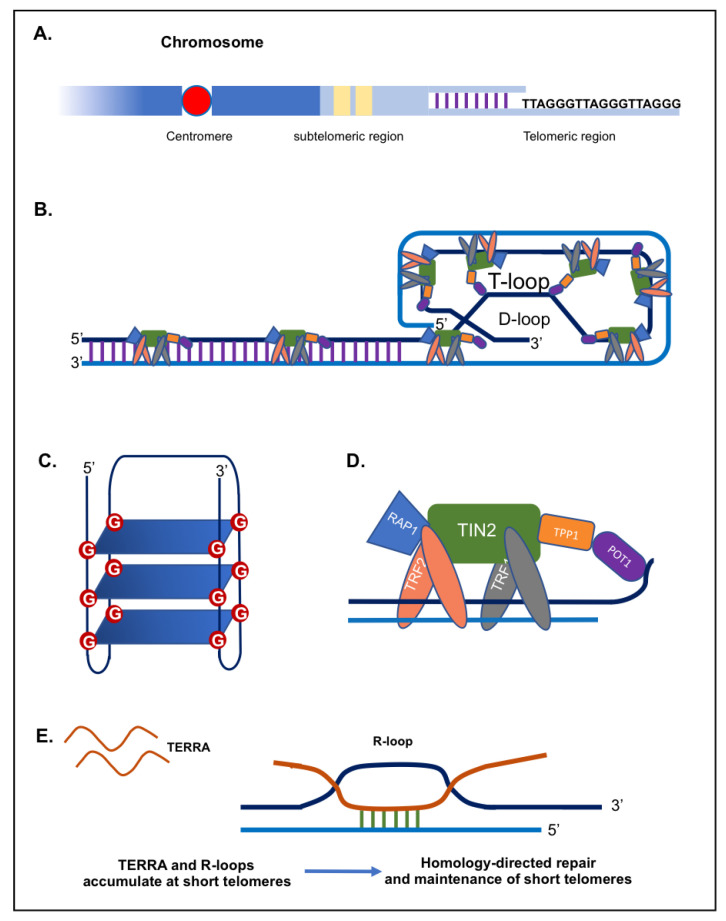
Telomeric DNA, proteins and TERRA. (**A**) Schematic representation of a telomeric region. (**B**) T-loop and D-loop configurations, along with the Shelterin proteins. (**C**) G-quadruplex structure. (**D**) The shelterin complex including the following proteins Telomeric-Repeat-binding Factor 1 and 2 (TRF1 and TRF2), Protection of Telomeres 1 (POT1), TRF1-Interacting Nuclear protein 2 (TIN2), TIN2-interacting protein (TPP1), and Repressor-Activator Protein 1 (RAP1). (**E**) TERRA and R-loops.

Telomeres are distinctive nucleoprotein structures found at the end of eukaryotic chromosomes. They play a major role in protecting chromosomes from degradation, fusion and erroneous recombination [1,2]. Telomeres comprise tandem repeats of a short DNA sequence (5′-(TTAAGGG)n-3′ in vertebrae) (Figure 1A) in addition to a terminal region consisting of a single-stranded G-rich 3′ overhang [3,4,5,6,7]. Telomere length varies considerably among species. For instance, *Mus musculus* have very long telomeres (20 to 50 kb), while in humans, telomeres are relatively short, comprising ~4 to ~15 kb of double-stranded repeats, ending in 50 to 400 nucleotides of single-stranded overhang [3,4,8]. Telomeric DNA can fold into higher-order structures to ensure the better protection of the chromosomal ends. The single stranded overhang folds back into the D-(displacement) loop, while the whole telomere (double stranded part) forms the T-(telomeric) loop (Figure 1B) [9,10,11]. Furthermore, G-rich telomeric repeats can fold into compact G-quadruplex structures involving the interaction of four guanine bases (G-quartet) in a square planar arrangement stabilized by hydrogen bonds (Figure 1C) [12,13]. 

Telomeric repeats are bound by a specialized set of telomere-binding proteins, known as “shelterin” complex, that are essential for chromosome integrity and stability, with crucial roles in telomere maintenance (Figure 1D) [14,15]. In human cells, the shelterin complex is made up of 6 core proteins: the TRF1 and TRF2 (telomeric repeat binding factor 1 and 2), the TIN2 (TRF1-interacting protein 2), POT1 (protection of telomeres protein 1), TPP1 (TIN2 and POT1 interacting protein), and the RAP1 (repressor/activator protein 1) (Figure 1D) [1,15,16]. First, TRF1 and TRF2 subunits bind directly to the double stranded DNA repeats. Then, both TRF1 and TRF2 interact with TIN2, which in turn links the heterodimer formed by TPP1 and POT1 to telomeres [17,18,19]. Finally, the subunit RAP1 binds to telomeres via an association with TRF2 [20,21]. Shelterin is considered as the main armour of chromosome ends, providing protection to the chromosomes and preventing chromosomal termini from activating a network of signalling cascades known as DNA damage response (DDR) [14,17]. Mainly, TRF2 and POT1 proteins enable the cell to distinguish natural chromosome ends from harmful double-stranded DNA breaks by preventing DNA damage and repair pathways from being activated [15,17,22]. It is worth noting that some proteins (i.e., nucleases and helicases) although present at the level of telomeres, do not belong to the shelterin complex, but act as accessory factors to the shelterin complex [14]. 

Because the ends of linear DNA cannot be replicated completely during lagging strand DNA synthesis, chromosome ends gradually shorten after every cell division, leading to what is called an “end replication problem” [23]. Consequently, the telomeres become short and dysfunctional, with a gradual loss of telomere-binding shelterin [14,24]. Short telomeres can be identified as DNA damaged sites, and the lack of shelterin proteins also leads to the activation of the DNA damage checkpoint [25,26,27]. In order to prevent telomere shortening, most eukaryotic cells express telomerase, an enzyme responsible for maintaining telomeres-length by adding telomeric repeats to the 3′ end of chromosomes [28]. In humans, the core of this ribonucleoprotein complex is composed of hTERT (human telomerase reverse transcriptase) and the telomerase RNA (TR) template subunit encoded by the telomerase RNA component (hTERC) gene. The role of this complex is to catalytically add telomeric repeats to the 3′ end of telomeres during the S phase [28,29]. In adults, in general, telomerase is expressed in proliferating cells (germ cells, stem cells, T-and B-lymphocytes and endothelial cells) and is silent in the majority of other somatic cells from early human development [30]. A significant reactivation of the telomerase is observed in most human cancer cells (~90%), which enables cancer cells to maintain and stabilize their telomere length, thus attaining unlimited proliferative capacity (proliferative immortality) [30,31]. The remaining ~10% of cancer cells maintain their telomeres through a different mechanism known as ALT (alternative lengthening of telomeres) [32,33]. Recent evidence suggests that ALT induction is driven by altered telomeric chromatin [34,35]. The progression from normal to cancerous cells involves various genetic and epigenetic events, occurring in several stages. These events not only allow cells to overcome proliferation barriers and escape the crisis that refers to the stage when further telomere erosion occur, leading to massive cell death and chromosomal instability, but also activate a telomere maintenance mechanism [36,37]. 

The activation of oncogenes and inhibition of tumour suppressor genes, along with a telomere maintenance mechanism, including several players such as telomeric proteins and/or telomerase (hTERT), may lead to cancer. Genetic and epigenetic modifications have been found to regulate *hTERT* expression, including promoter mutations (TERTp) [38,39] or methylation [40,41,42,43], and less frequently gene amplification or rearrangements [44,45,46]. Non-coding RNA (ncRNA), including micro-RNA (miRNA, miR) and long non-coding RNA (lncRNA) such as TERRA (telomeric repeat-containing RNA), have been reported to take part in telomeres and telomerase homeostasis and regulation [47,48]. Recent evidences indicate that TERRA molecules play a crucial role in telomere length regulation, telomerase activity, and heterochromatin formation at telomeres [49].

## 2. TERRA

Long ncRNAs “TERRA” stem from telomeres. They were reported to be transcribed for the first time in 1989 in the parasite Trypanosoma brucei [50]. Following this seminal report, several studies reported on TERRA in several species including humans [51,52]. Therefore, TERRA sequences are considered evolutionarily conserved in vertebrates [51,53]. Moreover, TERRA molecules are transcribed in a regulated manner from the subtelomeric regions towards chromosome ends by the enzyme RNA Polymerase II (RNA Pol II) using the C-rich strand as a template [47]. Although having a low gene density, the subtelomeric regions are nevertheless important for essential cellular activities such as those related to cell cycle regulation [54,55]. Several studies have used repeat-specific primers in PCR- and qPCR-based methods to analyse subtelomeric regions, and reported that TERRA is expressed from multiple chromosome ends in humans, such as 1q, 2q, 7p, 9p, 10q, 13q, 15p, 17p, 17q, 18q, 20q, XpYp, XqYq and others [56,57,58,59]. Therefore, using subtelomere-specific primers, the vast majority of studies so far have suggested that TERRA transcription occurs from subtelomeric promoters located on at least two-thirds of chromosome ends [60,61]. Furthermore, most cancer studies conducted on TERRA using telomerase-positive human cells, or ALT cancer cells, reported that TERRA are expressed as well from multiple chromosome ends [61,62,63]. TERRA are reportedly involved in several cellular processes and diseases [49,64,65]; recently, a preliminary study suggested, for the first time, a role for TERRA expression as a predictor of embryo quality in assisted reproduction [66].

TERRA length may range from 100nt to more than 9kb in mammals [51,67]. TERRA promoter regions comprise CpG islands, as well as binding sites for the CCCTC-binding factor (CTCF), which, along with Rad21, a component of the cohesin complex, regulate TERRA expression through binding to TERRA promoters in a number of human chromosomes [61,62,68]. Both CTCF and Rad21 promote the recruitment of RNA Pol II to the promoter region of TERRA allowing transcription [61]. Depletion of CTCF or Rad21 decreases TERRA levels and increases the activation of DDR at chromosome ends [61,68]. 

In human cells, the vast majority of TERRA contain 7-methylguanosine (m^7^G) cap structures at its 5′-ends, while a small fraction contain a poly(A) tail at its 3′-end, contributing to their stability [51,67]. Moreover, TERRA were detected at telomeres of freshly isolated human cells, suggesting that TERRA may bind to telomeres in vivo [60]. Furthermore, in human cells, TERRA expression was reported to vary in a cell-cycle-dependent manner, showing highest levels in G1 phase and gradually decreasing to lowest levels during S phase. An increase in expression is observed again at the end of cell division [67].

Several factors may interfere with, and in certain cases may limit TERRA detection and analysis, such as the methods and experimental protocols used, the number of passages for the cultured cells, as well as the genomic state of the analysed cells (high rates of rearrangements, telomere heterogeneity, etc). All of these may contribute to some of the observed discrepancies between different studies [65,69].

### 2.1. TERRA and Telomerase

Since the discovery of mammalian TERRA (telRNA) in 2008, it has been hypothesized that TERRA transcripts can regulate telomerase activity at chromosome ends in vitro [52,53]. Indeed, the 3′ end of TERRA is complementary to the telomerase RNA template region, although it is unclear whether TERRA binds the TERC template region [70]. In addition, TERRA has been reported as a telomerase ligand and a natural direct inhibitor of human telomerase [70]. Indeed, TERRA oligonucleotides have been shown to act as potent competitive inhibitors for the binding of telomeric DNA [70]. These pioneering studies have proven that TERRA can inhibit telomerase activity in vitro [52,70]. In another study on human cancer cells, TERRA overexpression had no effect on telomerase activity, challenging the previous hypothesis that TERRA works as a telomerase inhibitor [57]. 

TERRA are believed to bind the telomerase core components, TERT and TERC (or telomeric repeats), but the role of TERRA in telomerase function is still unclear [59]. Moreover, in yeast, TERRA expression is induced by telomere shortening and TERRA molecules act as a scaffold to promote telomerase nucleation and the formation of telomerase recruitment clusters [71,72]. As a result, TERRA interacts with yeast telomerase RNA *TLC1* (similar to *hTERC*) in vivo and forms TERRA-TLC1 complex which co-localizes with the telomere of origin during the S phase, suggesting a role for TERRA in the spatial organization of telomerase activity in telomeres [72].

In human cells, the interaction between TERRA and telomerase and the role of TERRA as a negative regulator “in vivo” remains unclear despite several models proposed [57,73]. Further advanced in vivo studies are needed to better understand the role of TERRA in the regulation of telomerase in human cells. 

### 2.2. TERRA, Telomeres and DNA Damage Response

The first report on the correlation between TERRA and telomere length was published in 2008 in patient cells affected by ICF (immunodeficiency, centromeric region instability, facial anomalies) syndrome, using an ectopic telomerase overexpression approach, in order to elongate the telomeres [74]. A few years later, using a similar approach in several human cell lines, it was demonstrated that TERRA is dependent on telomere length [75]. It was then established that telomere length may also impact TERRA expression levels, such that short telomeres display increased transcriptional activity which correlates with a decrease in repressive histone marks in telomeric repeats [75]. 

TERRA transcripts have been proven to promote proper assembly of telomere-binding proteins at chromosome ends (capping) and consequently preserve genomic integrity [76]. Moreover, TERRA transcripts have the capacity to function as epigenomic modulators in *trans* and as essential regulators of telomeres in *cis*, thus controlling DDR pathways indirectly by regulating gene expression [77]. Interestingly, TERRA transcripts have been shown to form hybrid structures (DNA-RNA) with the telomeric C-rich strand, named R-loops, which creates a permissive environment for the recombination of telomeric DNA (Figure 1E) [78,79]. In human cancer cells, higher levels of telomeric R-loops are detected in ALT-positive cancer cells, compared to telomerase-positive cancer cells [79]. Formation of R-loop structures at telomeres can impact telomere maintenance and genome stability through various mechanisms such as chromatin regulation and the promotion of homologous recombination between telomeres (Figure 1E) [80,81,82,83]. Indeed, R-loops can cause DNA damage and genome instability, most likely by interference with DNA polymerases during replication [84]. Therefore, TERRA can sustain telomeric DNA replication through R-loop formation at telomeres.

Moreover, TERRA transcripts have been proposed to contribute to proper telomere capping, which helps to prevent the activation of DDR at chromosome ends. In vitro, it was suggested that TERRA plays a role in switching at the ends of chromosomes between the DNA-binding protein RPA, required for the activation of the ATR checkpoint, and the shelterin component POT1 which acts as a telomeric repressor of the ATR-mediated DDR in telomeres [22,85]. This antagonist replacement is mediated by a direct interaction between TERRA and the RNA-binding protein hnRNPA1, which belongs to the hnRNPs family and which can regulate the abundance and localization of TERRA in telomeres [86].

The downregulation of TERRA (i.e., knockdown using siRNA) and an unscheduled accumulation of TERRA transcripts at telomeres both activate the DDR pathway at chromosome ends. As a consequence, an increase in telomere dysfunction-Induced Foci (TIFs) is observed, in addition to aberrations in metaphase telomeres [58,87]. Several experimental results have highlighted the important roles of TERRA expression, localization and tight regulation in maintaining chromosomal and telomeric stability [88]. Indeed, knocking out the TERRA locus from telomere 20q in U2OS cells, and depletion of TERRA expressed from telomere 18q in MEF (Mouse Embryonic Fibroblasts) cells result in DNA damage and telomere dysfunctions at multiple chromosome ends [58,59]. 

In addition to their role in controlling telomere ends, recent evidence has revealed that TERRA transcripts may also cause changes in the chromatin structure of uncapped telomeres [62].

### 2.3. TERRA and Chromatin Regulation

It has been demonstrated that compaction of chromatin mediated by trimethylation of H3K9 (H3K9me3) and H4K20 (H4K20me3), as well as telomeric DNA methylation could repress TERRA expression, resulting in discrepant expression levels of TERRA between normal and telomerase-positive cancer cells [64].

Emerging evidence has reported that TERRA transcription also plays a role in the regulation of heterochromatin formation at telomeres [65]. In particular, TERRA has been reported to promote the methylation of histone H3K9 and the accumulation of H3K9me3 in damaged telomeres, through interaction with the histone methyltransferase SUV39H1 upon depletion of the shelterin TRF2 [62]. Moreover, TERRA can actively participate in the DDR initiated by dysfunctional telomeres by promoting the association of several enzymes, including chromatin remodelling factors at telomeres [62]. So, TERRA transcripts can interact with heterochromatic marks (H3K9me3 and HP1 proteins), histone methyltransferase SUV39H1 and with chromatin remodelling complexes such as NoRC (nucleolar remodeling complex), MORF4L2 (a component of the NuA histone acetyltransferase complex), and ARID1A (a component of the SWI/SNF nucleosome remodeling complex) [65,87,89]. Recent studies showed that TERRA can associate with the histone methyl transferase PRC2 (Polycomb Repressive Complex 2), through direct interaction with EZH2 and SUZ12, both components of PRC2 [90].

ALT-positive tumour cells express the highest levels of TERRA, likely due to open chromatin conformation at subtelomeres and to abnormally long telomeres resulting from an adaptive response to mutations in *ATRX* or *DAXX* genes, both considered chromatin remodelling factors [91].

### 2.4. TERRA and Stress

Studies exploring the interactions between TERRA, DDR and cellular stress have demonstrated that heat shock (heat stress) may promote the accumulation of TERRA in mouse and human cells [52,92]. It has been recently reported that, in response to heat stress, the transcription factor HSF1 (heat shock factor 1) binds to telomeres and triggers TERRA accumulation, thus protecting the telomeres from TIF accumulation caused by exposure to stress [93].

In an attempt to induce TERRA transcription, Tutton et al. used etoposide (chemotherapeutic agent) treatment or serum-free medium for cell cultures in HCT116 cell line [94]; they additionally used the genome editing tool CRISPR in the same stable HCT116 cell line to delete the p53-binding site on chromosome 18q, which resulted in a decrease in p53-responsiveness of the subtelomeric transcript. This showed that DNA damage stress may induce 18q-specific TERRA transcription in a p53-dependent manner [94]. The findings also suggest that p53 contributes to the stress-induced activation of subtelomeric and TERRA-like transcripts from multiple chromosomes [94]. 

Furthermore, it was reported that TERRA may play a role in the response to oxidative stress, and thus may take part in the cellular metabolism process. Indeed, during endurance exercises in human muscle tissue, TERRA expression is upregulated by the antioxidant transcription factor NRF1 (nuclear respiratory factor 1), and is triggered by the telomeres [60]. TERRA may as well be triggered by oxidative stress; and may be regulated by PKA (Protein kinase A) signalling, as well as by changes in the cytoskeleton dynamics [95].

Altogether, these findings suggest that TERRA induction may play an important role in preserving telomeres integrity during cellular stress. Further research on TERRA transcription regulation during physiological and pathological stress is still needed.

### 2.5. TERRA and Aging

Growing evidence supports an association between TERRA expression and the ageing process [96]. It has been shown that ICF syndrome patient cells (type I) have accelerated telomere shortening and loss; they therefore enter prematurely into replicative senescence and display high TERRA levels [97].

According to the “subtelomere–telomere” theory, where the repression of important regulatory sequences represents a pivotal part of the ageing mechanisms, TERRA sequences and transcripts could constitute an essential trigger in the onset of senescence and the ageing process [98]. Indeed, the role of TERRA clearly underlines its implication in genetically modulated mechanisms that could determine aging, however this still needs to be corroborated by further studies [98].

### 2.6. TERRA and Cancer

Telomerase or ALT activation stabilizes telomere length in cancer cells; nevertheless, shelterin complex aberrations as well as alterations in TERRA expression are frequently reported during oncogenesis. These alterations may impact telomeric chromatin, in a direct or indirect way, promoting tumour initiation and/or progression [64]. A recent study on human endometrial carcinogenesis analysing TERRA levels from multiple chromosome ends (1q-2q-4q-10q-13q-22q, 16p and 20q) have shown a negative correlation between the expression levels of two TERRA (16p and 20q) and the proliferative marker Ki67, suggesting a role for TERRAs in carcinogenesis [99].

Some telomerase-positive cancers such as advanced stages of the larynx, stomach, colon cancer, and lymph node tumours, do not show high expression levels of TERRA when compared to normal tissue [57]. On the other hand, TERRA levels were reportedly elevated in tumours with long telomeres, lacking telomerase activity and ALT-positive. [73]. A recent study reported significantly variable levels of TERRA among ALT-positive cells, suggesting that several epigenetic modifications could activate telomeric recombination in ALT-positive cells. These results may have an important impact on the therapeutic approaches needed to overcome ALT-dependent cellular immortalization [100]. In different studies, it was demonstrated that some cancer cells exhibit lower TERRA expression [99]. Altogether, these data emphasize the broad variability of changes in TERRA expression observed in different cancer types.

Moreover, as previously mentioned, telomerase-negative ALT-positive cancer cells present higher levels of R-loops at their telomeres [79]. It has been recently reported that BRCA1 (Breast cancer gene 1) binds TERRA RNA as well as telomere-specific shelterin proteins in the R-loop, and in a cell cycle-dependent manner, suggesting that normal BRCA1/TERRA binding suppresses telomeric genome instability [101]. Additionally, BRCA2 deletion was reported to trigger TERRA overexpression and ALT mechanisms in colon cancer cells in the presence of telomerase activity [102]. These findings open several questions about BRCA-mutated cancers and anti-telomerase therapies.

Accordingly, TERRA involvement in several cellular processes, such as directing the proper assembly of the shelterin, facilitating telomeric DNA replication, promoting DNA damage response and repair at dysfunctional telomeres and heterochromatin formation at subtelomeric and telomeric regions, suggests that targeting TERRA could be a potential strategy to target tumour cells and therefore could represent a new opportunity for cancer therapy [59,64].

Numerous epigenetic alterations were reported in lymphoma cell lines and patient cells. These alterations are considered biomarkers that contribute to lymphomagenesis; including enzymes responsible for DNA methylation, histone modifications as well as non-coding RNA [103].

### 2.7. TERRA in Cutaneous T-Cell Lymphomas

Cutaneous T-cell lymphomas (CTCL) are non-Hodgkin lymphomas, encompassing a heterogeneous group of rare T lymphoproliferative disorders (cutaneous anaplastic large cell lymphoma (C-ALCL), mycosis fungoïdes (MF), Sézary syndrome (SS) and others). They are characterized by clonal proliferation of malignant T lymphocytes stemming from the skin [104]. Treatment of advanced-stage MF/SS can be very challenging; therefore, there is still a need for innovative therapies and the identification of new targetable biomarkers [105]. In a pioneer investigation, our team explored the telomerase status and telomeres length in CTCL. First, we reported that CTCL are telomerase-positive tumours, with the shortest telomeres observed in the aggressive forms of the disease [44,106]. Our results confirmed Wu et al.’s observation showing that CTCL cells exhibit short telomeres [107]. We subsequently demonstrated that in this pathology, DNA methylation is associated with telomerase (*hTERT*) expression [42,108]. Therefore, we performed further epigenomic investigations in CTCL cells, focusing on telomeres and telomerase. To our knowledge, the data described below represents the first report on TERRA in CTCL. 

## 3. Materials and Methods

### 3.1. Biological Material

**Figure 2:**
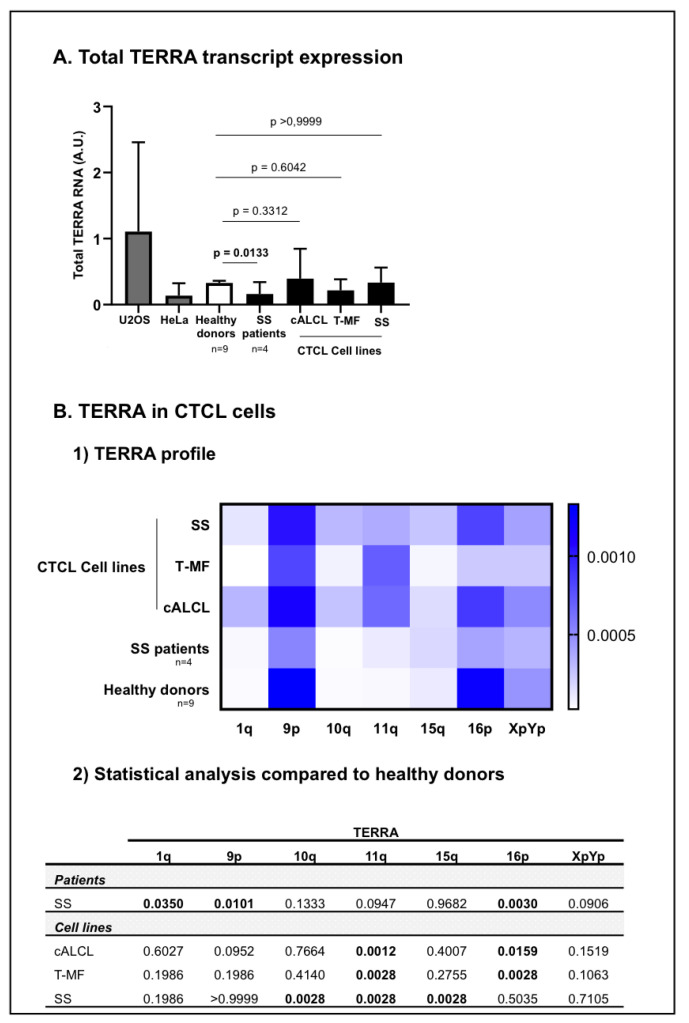
Telomeric repeat containing RNA (TERRA) in CTCL cells. TERRA from chromosome ends 1q, 9p, 10q, 11q, 15q, 16p and XpYp were analysed in CTCL cells, including SS patients (n = 4) and CTCL cell lines representative of different CTCL subtypes: c-ALCL, T-MF, and SS), and then compared with healthy donors (n = 9). (**A**) The total TERRA expression was evaluated. Before the analysis of our cells of interest, the RT-qPCR protocol for quantification of TERRA was validated in HeLa and U2OS cell lines (in grey). We confirmed that U2OS and HeLa cells express different TERRA levels [56]. Data are expressed as mean ± SD. (**B**) For CTCL cells we presented the (1) TERRA profile for seven telomeric transcripts in a heat map: horizontal axis = TERRA transcripts; vertical axis = cells analysed. Low expression levels are displayed in light blue, and high expression levels in dark blue. (2) Statistical analysis from TERRA profiling. Significance level: *p* = 0.05.

Experiments were performed in five CTCL cell lines, including three cALCL: Mac1, Mac2A, Mac2B (DSMZ), one transformed MF (T-MF): My-La (Dr K. Kaltoft, Aarhus, Denmark), and one SS: Hut78 (ATCC). Furthermore, U20S (ATCC), an osteosarcoma cell line and HeLa (ATCC), a cervical cancer cell line, were used as positive controls for TERRA amplification (Figure 2). CTCL cell lines were cultured in RPMI 1640 media (Gibco) supplemented with 100 U/mL penicillin, 100 μg/mL streptomycin (Gibco) and 10% foetal bovine serum (Eurobio). U2OS and HeLa were cultured in DMEM (Gibco), supplemented with 100 U/mL penicillin (Gibco), 100 µg/mL streptomycin (Gibco) and 10% foetal bovine serum (Eurobio). All cell lines were incubated at 37 °C with 5% CO_2_ and regularly tested for Mycoplasma contamination.

Sézary Syndrome patients (n = 4) were selected from the dermatology department, Bordeaux University Hospital Centre (CHU) (France), according to the criteria of the World Health Organization-European Organization for Research and Treatment of Cancer (WHO-EORTC) [104]. The institutional review board approved the manipulation of CTCL patients’ samples (DC-2015-412). Peripheral blood from healthy individuals (n = 9) were obtained from the Etablissement Français du Sang (EFS), France (DC 2015 2412-18PLER012). Peripheral blood mononuclear cells from patients and healthy donors were isolated using PANCOLL^®^ density gradient centrifugation (PAN-Biotech, Aidenbach, Germany).

### 3.2. TERRA Quantification

Total RNA was isolated using Direct-zol™ RNA MiniPrep kit (ZYMO Research). All RNAs were treated with DNAse Max^TM^ kit (Quiagen), according to the manufacturer’s instructions. Using specific anti-sense primers for each target, 3 µg of ARN was reverse transcribed using SuperScript II reverse transcriptase kit (Invitrogen, Waltham, MA, USA), according to the manufacturer’s instructions. Complementary DNA (cDNA) was amplified by quantitative reverse-transcription PCR (qRT-PCR), using Takyon^TM^ No Rox SYBR^®^ MasterMix dTTP Blue (Eurogentec, Seraing, Belgium) and specific primers. qRT-PCR analyses were run on a Stratagene Mx3005P system (Agilent Technologies, Santa Clara, CA, USA) and analysed with MxPro 4.01 qPCR software Stratagene (Agilent Technologies, Santa Clara, CA, USA). Data was normalized using the Human elongation factor-1 *α* (EF-1*α*). Data analysis was performed using the 2^−∆CT^ method. Total TERRA was calculated as the sum of analysed TERRA from selected chromosomes. Sequences of all primers used are available in Supplementary Table S1 [56,67,75].

### 3.3. hTERT Overexpression

Telomerase overexpression was performed as previously reported [44]. HuT78 cells were seeded into 6-well plates and then transduced, either with a lentiviral vector containing *hTERT* complementary DNA or a lentiviral vector containing DsRed2 (used as control), as previously published [44]. Transduced cells were selected with puromycin and expanded in 6-well plates until an appropriate cell growth rate was achieved. *hTERT* overexpression was confirmed by RT-qPCR (Supplementary Figure S1), and cells were transferred to culture flasks for further expansion. Cells were cultured and monitored over a period of two months (~20 passages). More details regarding the technique are available in the methods and supplementary methods of the following article [44].

### 3.4. Telomerase Activity Estimation

Telomerase activity was assessed from protein extracts using MilliporeSigma™ Chemicon™ TRAPeze™ RT telomerase detection kit (Fisher scientific, Hampton, NH, USA), according to manufacturer’s instructions with minor modifications, as previously described [44].

### 3.5. Telomere Length Estimation

Genomic DNA was extracted by the salt precipitation method, as previously described [44]. The Absolute Human Telomere Length Quantification qPCR Assay Kit (CliniSciences, Nanterre, France) and FastStart Essential DNA Green Master (Roche, Mannheim, Germany) were used to estimate telomere length.

### 3.6. Statistics

Statistical analyses were conducted using GraphPad Prism software (version 8.0.1). Data from patients were collected from duplicate reactions for each sample. Data from cell lines were collected from triplicate reactions from four independent experiments. Data from transduced cell lines were collected from duplicate reactions from three independent experiments. The “n” values consist only of independent biological repeats and technical repeats that have been averaged before. The results are represented as mean ± standard deviation. Paired Mann–Whitney test (nonparametric *T*-test) was used to compare variables. The significance level was set to *p* = 0.05.

## 4. Results

### 4.1. CTCL Telomeric Regions Are Transcribed

TERRA from telomeric chromosomes 1q, 9p, 10q, 11q, 15q, 16p and XpYp were quantified in positive controls in healthy donors and in CTCL cells (Figure 2). As expected, U2OS cells tend to express high levels of TERRA and Hela cells express low levels of TERRA [56] (Figure 2A) (individual TERRA: Supplementary Figure S2).

In healthy donor cells, among TERRA transcripts screened, TERRA 9p and 16p were the most expressed, while TERRA 1q, 10q, 11q and 15q were the least expressed (Figure 2B). Cell lines representative of the most common CTCL subtypes: cALCL, T-MF and SS, as well as SS patient cells were analysed and compared with healthy donor cells. In CTCL cells, TERRA 9p, 11q and 16p were the most expressed and TERRA 1q, 10q and 15q were the least expressed (Figure 2B). At first glance, the CTCL cell lines did not express any statistical differences in the total amounts TERRA when compared to healthy donors (Figure 2A). Nevertheless, cALCL cell lines exhibited a significant increase in TERRA 11q (*p* = 0.0012) and a significant decrease in TERRA 16p (*p* = 0.0159) (Figure 1(B1) and Figure 2(B2)). Similarly, the T-MF cell line showed a significant increase in TERRA 11q (*p* = 0.0028) and a significant decrease in TERRA 16p (*p* = 0.0028) (Figure 1B and Figure 2(B2)). Additionally, in the SS cell line, Hut78, we observed a significant increase in TERRA 10q (*p* = 0.0028), 11q (*p* = 0.0028) and 15q (*p* = 0.0028) compared to healthy donors’ cells (Figure 2(B1,B2)). As for SS patient cells, they expressed significantly less total TERRA amounts than healthy donor cells (*p* = 0.0133) (Figure 2A). Compared to healthy donors’ cells, we observed a significant increase in TERRA 1q (*p* = 0.0350), along with a significant decrease in TERRA 9p (*p* = 0.0101) and 16p (*p* = 0.0030) in Sézary patient cells (Figure 2(B1,B2)) (Supplementary Figure S3). 

### 4.2. hTERT Overexpression Downregulates TERRA Transcripts

**Figure 3:**
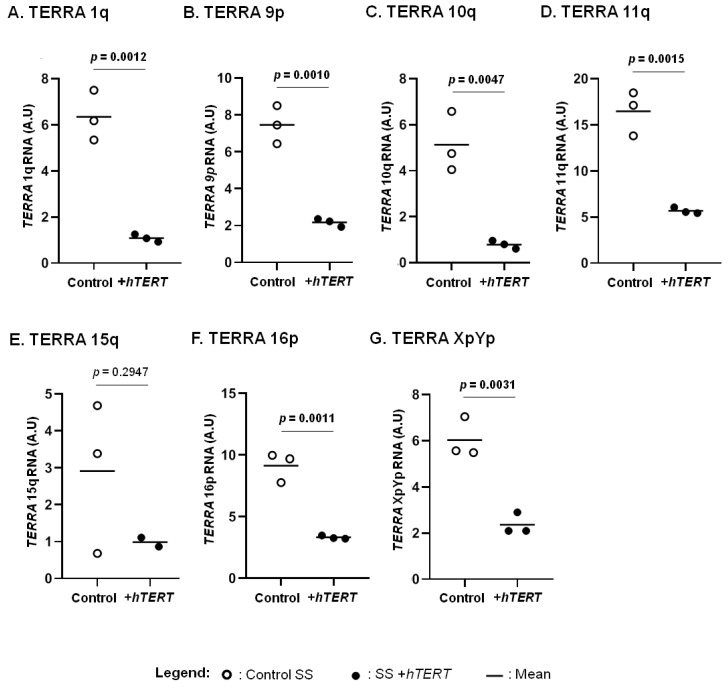
*hTERT* impact on TERRA transcription. The expression of TERRA from chromosome ends 1q (**A**), 9p (**B**), 10q (**C**), 11q (**D**), 15q (**E**), 16p (**F**) and XpYp (**G**) were analysed in T-MF and SS cells overexpressing *hTERT* and in their respective controls. Statistical analysis results are displayed on each graph. Significance level: *p* = 0.05. Data are expressed as mean ± SD.

We previously reported a correlation between *hTERT* expression and aggressive forms of CTCL [44]. Here, we focused on the HuT78 cell line, representative of SS, an aggressive CTCL subtype (Figure 3). We assessed the expression of TERRA in CTCL cells overexpressing *hTERT,* in comparison with their respective controls. A significant decrease in all TERRA analysed was observed in hTERT transduced cell line, when compared to controls, except for TERRA 15q (Figure 3).

### 4.3. TERRA Negatively Correlates with Telomere Length in SS Cells

**Figure 4:**
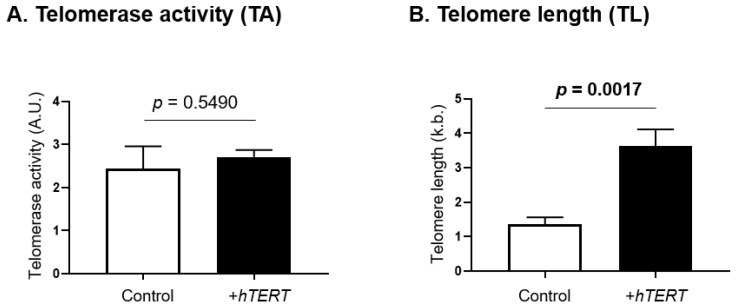
Functional impact of *hTERT* overexpression on telomerase activity and telomere length. Telomerase canonical functions were evaluated in SS cells overexpressing hTERT, through the assessment of (**A**) telomerase activity and (**B**) telomere length. Statistical analysis results are displayed on each graph. Significance level: *p* = 0.05.

Telomerase activity and telomere length were assessed in SS cells overexpressing *hTERT* and compared with their respective controls (Figure 4). While *hTERT* overexpression had no impact on telomerase activity (Figure 4A), a significant increase in telomere length (*p* = 0.0017) was observed (Figure 4B).

## 5. Discussion

In order to unveil new potentially targetable biomarkers for diagnostic and follow-up of CTCL, we conducted a pilot investigation on the involvement of long non-coding RNA molecules (TERRA) in CTCL.

We studied TERRA from chromosome ends 1q, 9p, 10q, 11q, 15q, 16p and XpYp. In order to obtain reference values, we first analysed TERRA expression in lymphocytes from nine healthy donors. We found that while TERRA 1q, 10q and 11q were poorly expressed, TERRA 9p and 16p were highly expressed. Once our reference values were established, we were able to compare them to the data obtained in tumour cells, whether derived from cell lines or from patients.

We were able to demonstrate that in CTCL, like in any other type of cancer [57,99], TERRA stems from multiple telomeric chromosomal ends. TERRA levels in CTCL cells, which are telomerase-positive cells, are greatly reduced compared to healthy controls and cells engaged in ALT mechanism [65]. Moreover, the three CTCL subtypes analysed (cALCL, T-MF and SS) presented with different profiles of TERRA transcripts, each distinct from the profile presented by the controls. This suggests that each CTCL subtype has a peculiar TERRA signature. Nevertheless, among all cells analysed (CTCL cells and healthy controls), TERRA 9p and 16p were the most commonly expressed, while TERRA 1q, 10q and 15q were less expressed. Interestingly, TERRA 16p was downregulated in all CTCL samples (patients and cell lines) compared to healthy controls; a similar observation has been reported in endometrial cancer [99]. In CTCL, we noticed that this low expression was more pronounced in aggressive CTCL subtypes (T-MF cell line and SS patients’ cells). These findings suggest the potential involvement of a TERRA 16p decrease in disease aggressiveness. Moreover, our data show that TERRA 11q is among the most commonly expressed TERRA in CTCL cells (cell lines and patient cells), while it is among the least expressed in healthy cells. In general, in the literature, it is stated that TERRA expression is not elevated in telomerase-positive cancer cells [57], but here we report that telomerase-positive CTCL cells display a specific TERRA expression profile and support the potential involvement of TERRA 11q and 16p in CTCL biology. 

TERRA have been implicated in telomerase activity, as well as the regulation of telomere length [65]. In a previous work, we reported that *hTERT* overexpression stimulates proliferation as well as tumorigenesis in vitro and in vivo [44], and here we showed that *hTERT* overexpression also downregulates global TERRA expression. Furthermore, our results show that, although *hTERT* overexpression does not impact telomerase activity, it induces significant telomere lengthening over time, confirming that other mechanisms such as changes in telomere accessibility by telomerase are possible. Thus, while TERRA transcripts were believed to inhibit telomerase activity [70], new studies show a stimulation of telomerase activity [109]. Our results suggest that TERRA may be involved in the regulation of canonical telomerase functions in SS cells.

## 6. Conclusions

Investigating TERRA transcripts opens a new era in understanding telomere functions and can provide valuable insights into telomere biology. Our results support the involvement of specific TERRA in CTCL and a possible role as a regulator of canonical telomerase functions. Before considering TERRA as a therapeutic target, our seminal results should be confirmed in additional CTCL cell lines and patients’ samples and reinforced by functional studies in order to decipher the precise molecular role of TERRA in the development of CTCL and disease progression.

## Data Availability

Not applicable.

## Supplementary Materials

The following supporting information can be downloaded at: https://www.mdpi.com/article/10.3390/genes13030539/s1, Figure S1: *hTERT* expression in transduced SS cells. Figure S2. TERRA levels in HeLa and U2OS. Figure S3. *hTERT* impact on TERRA. Supplementary Table S1. Primers sequences used for TERRA amplification.

## Abbreviations

ALT	alternative lengthening of telomeres
C-ALCL	cutaneous anaplastic large cell lymphoma
CTCF	CCCTC-binding factor
CTCL	cutaneous T-cell lymphomas
DDR	DNA damage response
hTERC	human telomerase RNA gene
hTERT	human telomerase reverse transcriptase
MF	mycosis fungoïdes
POT1	protection of telomeres protein 1
RAP1	repressor/activator protein 1
SS	Sézary syndrome
TERRA	telomeric repeat-containing RNA
TIN2	TRF1-interacting protein 2
T-MF	transformed mycosis fungoïdes
TPP1	TIN2 and POT1 interacting protein
TRF1	telomeric repeat binding factor 1
TRF2	telomeric repeat binding factor 2
