# Supplementary material for: Telomeric Repeat-Containing RNA (TERRA): A Review of the Literature and First Assessment in Cutaneous T-Cell Lymphomas
Source: Genes (Basel). 2022 Mar 18;13(3):539. doi: 10.3390/genes13030539 (PMC8953746; doi:10.3390/genes13030539)
Supplement: Supplementary file 1 [file genes-13-00539-s001.zip › genes-1623893-supplementary.pdf]

SUPPLEMENTARY DATA

Supplementary Figures:

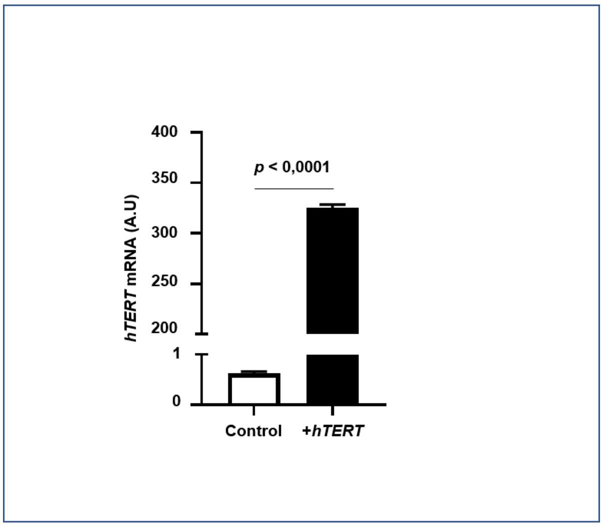

Supplementary Figure S1. *hTERT* expression in transduced SS cells. The expression of *hTERT* was assessed in transduced SS cells by RT-qPCR.

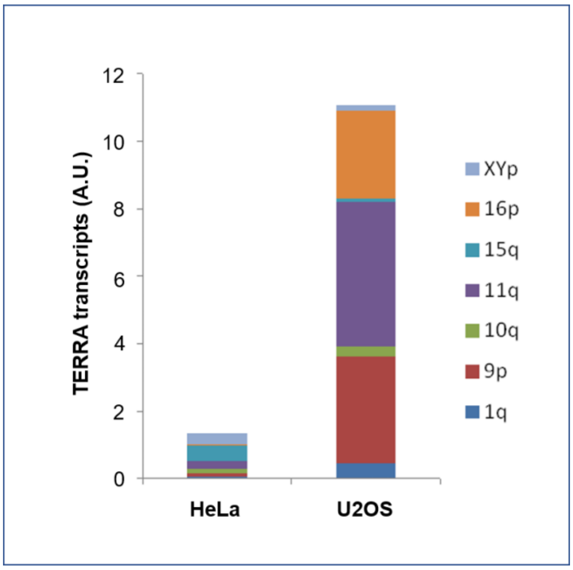

Supplementary Figure S2. ***TERRA levels in HeLa and U2OS.*** The detailed individual expression of TERRA in the control cell lines.

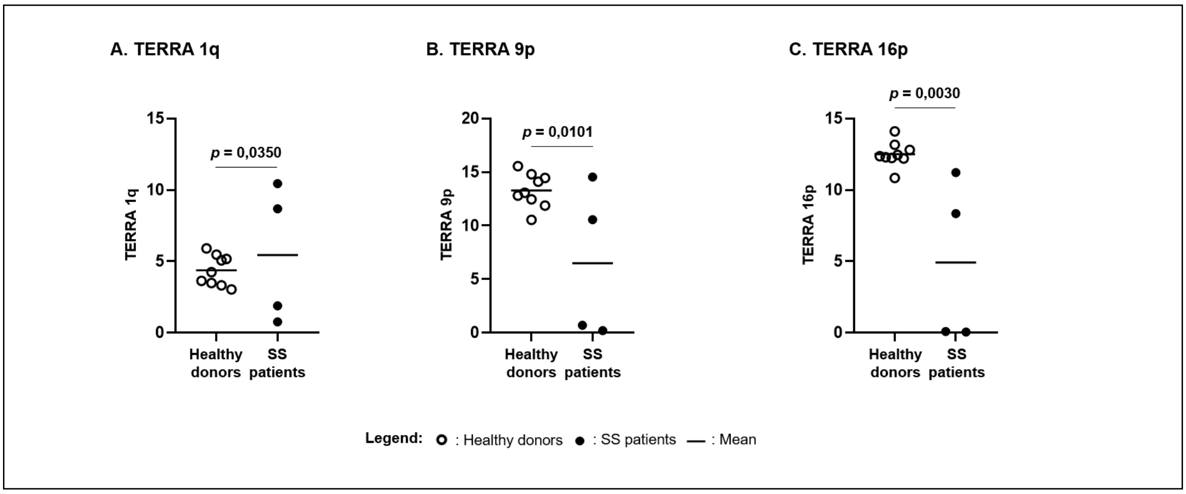

Supplementary Figure S3. ***hTERT* impact on TERRA.** The expression of TERRA from chromosome ends 1q, 9p and 16p were analysed in SS patients and in healthy controls. Statistical analysis results are displayed on each graph. Significance level: $p = 0.05$.

Supplementary Table S1. Primers sequences used for TERRA amplification**.**

Primers	Primer sequence
**TERRA 1q** (56)	
Fw	5'-GCATTCCTAATGCACACATGAC-3'
Rv	5'-ACCCTAACCCGAACCCTA-3'
**TERRA 9p** (56)	
Fw	5'-GAGATTCTCCCAAGGCAAGG-3'
Rv	5'-ACATGAGGAATGTGGGTGTTAT-3'
**TERRA 10q** (56)	
Fw	5'-ATGCACACATGACACCCTAAA-3'
Rv	5'-TACCCGAACCTGAACCCTAA-3'
**TERRA 11q** (75)	
Fw	5'-CTGATTATTCAGGGCTGCAAA-3'
Rv	5'-GCCGCATCGACGGTGAATAA-3'
**TERRA 15q** (67)	
Fw	5'-CAGCGAGATTCTCCCAAGCTAAG-3'
Rv	5'-AACCCTAACCACATGAGCAACG-3'
**TERRA 16p** (75)	
Fw	5'-TGTGTTTCAACGCTGCAACTG-3'
Rv	5'-AGTTAGAACGGTTCAGTGTG-3'
**TERRA Xp-Yp** (67)	
Fw	5'-GCAAAGAGTGAAAGAACGAAGCTT-3'
Rv	5'-CCCTCTGAAAGTGGACCAATCA-3'
**EF1α**	
Fw	5'-CTGGAGCCAAGTGCTAACATG-3'
Rv	5'-CCGGGTTTGAGAACACCAGT-3'
